# Heritability of cardiovascular risk factors in a Brazilian population: Baependi Heart Study

**DOI:** 10.1186/1471-2350-9-32

**Published:** 2008-04-22

**Authors:** Camila M de Oliveira, Alexandre C Pereira, Mariza de Andrade, Júlia M Soler, José E Krieger

**Affiliations:** 1 Laboratory of Genetics and Molecular Cardiology, Heart Institute, University of Sao Paulo Medical School, Sao Paulo, Brazil; 2 Division of Biostatistics, Department of Health Sciences Research, Mayo Clinic College of Medicine, Rochester, Minnesota, USA; 3 Statistics Department, University of Sao Paulo, Sao Paulo, Brazil

## Abstract

**Background:**

The heritability of cardiovascular risk factors is expected to differ between populations because of the different distribution of environmental risk factors, as well as the genetic make-up of different human populations.

**Methods:**

The purpose of this analysis was to evaluate genetic and environmental influences on cardiovascular risk factor traits, using a variance component approach, by estimating the heritability of these traits in a sample of 1,666 individuals in 81 families ascertained randomly from a highly admixed population of a city in a rural area in Brazil.

**Results:**

Before adjustment for sex, age, age^2^, and age × sex interaction, polygenic heritability of systolic (SBP) and diastolic (DBP) blood pressure were 15.0% and 16.4%, waist circumference 26.1%, triglycerides 25.7%, fasting glucose 32.8%, HDL-c 31.2%, total cholesterol 28.6%, LDL-c 26.3%, BMI 39.1%. Adjustment for covariates increased polygenic heritability estimates for all traits mainly systolic and diastolic blood pressure (25.9 and 26.2%, respectively), waist circumference (40.1%), and BMI (51.0%).

**Conclusion:**

Heritability estimates for cardiovascular traits in the Brazilian population are high and not significantly different from other studied worldwide populations. Mapping efforts to identify genetic loci associated with variability of these traits are warranted.

## Background

The etiology of cardiovascular disease is complex and it is thought to involve metabolic, neuro-endocrine and genetic interactions [1-4]. Prospective twin studies [5], familial aggregation [6] and intercorrelation analyses [7] have supported the existence of significant genetic influence modulating inter-individual variability for cardiovascular related traits. However, results of genetic epidemiological studies have been inconsistent, and it is unknown whether the genetic effect occurs through a major locus [4,8] or multiple [2,4] distinct loci acting in concert, but with relatively small effects.

The heritability of cardiovascular risk factors is expected to differ between populations because of the different distribution of environmental risk factors, as well as the particular genetic make-up of different human populations. Because of the high prevalence of cardiovascular disease and its risk factors, many studies have been conducted in populations worldwide to estimate their heritability. These studies have presented conflicting results probably due to different populations being used to derive these inferences (3, 4), such as populations with high prevalence of cardiovascular disease (16,18), diabetes (7) and obesity (7), or healthy populations (12,13,25). Thus, the purpose of this study was to evaluate genetic and environmental influences on cardiovascular risk factor traits, using a variance component approach, by estimating the heritability of these traits in a sample of extended pedigrees ascertained from a highly admixed population of a rural city in Brazil.

## Methods

### Study population and sample design

Between December 2005 and January 2006, 119 families (1,712 individuals) were selected in Baependi, a city in a rural area (752 Km^2^, 18,072 inhabitants) located in Minas Gerais State, Brazil. Probands were identified from the community at large in several stages. First, eleven census districts (from a total of twelve) were selected for study. Second, residential addresses within each district were randomly selected (first by randomly selecting a street, second a household). Finally, eligibility criteria (any individual living in the selected household who was 18 years old or above) within each household were established.

Once a proband was enrolled, all his/her first-degree (eg, parents, siblings, and offspring), second-degree (eg, half-siblings, grandparents/grandchildren, aunts/uncles, nieces/nephews, and double cousins), and third-degree (eg, first cousins, great-uncles/great-aunts, and great-nephews/great-nieces) relatives and his/her respective spouse's relatives, who were at least 18 years old, were invited to participate. After the proband's first contact, first degree relatives were invited to participate by phone; these included all living relatives in the city of Baependi (urban and rural area) and surrounding cities. To recruit the participants, the project was advertised through provincial, religious, and municipal authorities, in local television, newspaper, and radio messages, through physicians, and by phone calls. For physical examination, a clinic was established in a quiet but easily accessible sector of Baependi. Only individuals age 18 and older were eligible to participate in the study.

### Trait description

A questionnaire was administered to each participant to obtain information on family relationships, demographic characteristics, medical history, and environmental risk factors such as smoking habit, alcohol use, physical activity, and prescription drug use (e.g., anti-hypertensive, for diabetes, for dyslipidemia). Questionnaire was administered and filled out by research assistants specially trained for this task. The questionnaire was based on the WHO-MONICA epidemiological instrument and was previously used by our group in other epidemiological projects [9,10].

Anthropometric measures such as weight, body height and waist circumference were measured following standardized procedures. Height was measured in centimeters and weight in kilograms using a calibrated digital balance. Body mass index (BMI) (weight in Kg/height in meters^2^) was calculated and overweight defined as BMI ≥ 25 Kg/m^2 ^or obesity, BMI ≥ 30 Kg/m^2^. Waist circumference (WC) was measured half way between the lowest rib and the iliac crest while the subject was at minimal respiration. An individual was considered sedentary who worked seated or did not walk during work and who did not have any physical activity during leisure time. Smoking status was defined as "current smoker" when smoking has occurred during the last six months. Blood pressure was measured using a standard digital sphygmomanometer (OMRON, Brazil) on the left arm after 5 minutes rest, in the sitting position. Systolic and diastolic blood pressures were calculated from three readings (mean value of all measurements), with a minimal interval of 3 minutes. Physical examination and electrocardiogram were performed concurrently by trained medical students.

Fasting blood glucose, total cholesterol, lipoprotein fractions, and triglycerides were assayed by standard techniques in 12 hour fasting blood samples. Serum samples were stored at -80°C and genomic DNA was extracted following a standard salting-out procedure.

The study protocol was approved by the ethics committee of the *Hospital das Clinicas*, University of Sao Paulo, Brazil, and each subject provided informed written consent before participation.

### Statistical Analysis

Descriptive statistics were calculated to describe familial structure of the Baependi data set. In addition, descriptive measures, such as mean, median, standard deviation, skewness and kurtosis, were calculated for all traits considering the total sample and gender stratification. When the normality assumption did not hold for a specific trait, natural log-transformation was applied followed by a new data assessment. After the trait transformations, when the residual kurtosis remained too high we tried to prevent biased heritability estimates using robust estimation implemented in SOLAR through the *tdist *procedure.

Polygenic heritability estimates were calculated, adjusted for age, gender and medication use, for each cardiovascular related trait, using the variance-components approach implemented in the SOLAR package [11]. Heritability was calculated as the proportion of the total phenotypic variance explained by additive genetic effects after accounting for covariates.

The overall aim of these analyses was to determine the extent to which unmeasured genetic factors, and measured environmental and lifestyle factors, contributed to variation in a large panel of cardiovascular-related traits. Information on the covariance among relatives was used to estimate the polygenic (or additive genetic) component of variance. The variance component model is a well-known tool for heritability estimates in family studies and is only briefly described here [11,12]. Under this model, the level of the trait for individual *I *(denoted by *y*_*i*_) is described as follows:

$$y_{i}=\mu +\sum_{j=1}^{c}\beta_{j}X_{ij}+g_{i}+e_{i}$$

where *μ *is the general mean of the trait, and *β*_*j *_is the regression coefficient for covariate *j*, which assumes the value *X*_*ij *_for individual *i*. Measures of the qualitative covariates (eg, female gender, diabetes, medication use, etc.) were scaled so that the regression coefficient represents the effect of having the covariate present compared with having it absent. The remaining parameters, *g*_*i *_and *e*_*i *_are the residual genetic effect due the polygenic term, and random error component, respectively. The random effects, *g*_*i *_and *e*_*i *_are assumed to be uncorrelated and normally distributed with mean zero and variance $\sigma_{g}^{2}$ and $\sigma_{e}^{2}$ respectively. As usual, the error component is unique to each individual, whereas the polygenic component is shared between individuals in proportion to their kinship coefficient. Thus, the covariance between traits for individuals *i *and *i*' is given by:

$$Cov\left(y_{i};y_{i^{\prime }}\right)=\{\begin{array}{c} \begin{array}{ccc} \sigma_{g}^{2}+\sigma_{e}^{2} & for & i=i', \end{array}\\ \begin{array}{ccc} 2\varphi_{ii'}\sigma_{g}^{2} & for & i\neq i',but\;related, \end{array}\\ \begin{array}{ccccc} 0 & for & i\neq i' & and & unrelated \end{array}. \end{array}$$

Parameter 2*φ*_*ii *_is the coefficient of relationship between individuals *i *and *i*^'^. The likelihood of the traits of family members is assumed to follow a multivariate normal distribution. Estimates of the mean and variance components are obtained using maximum likelihood methods.

Firstly the variance component model was fitted without any covariate effects, denoted by unadjusted model (Model 1). Secondly two sets of covariate effects on each traits were considered: under Model 2 the covariates were sex, age, age^2^, and sex × age interaction; under Model 3, besides the covariates from Model 2, current medication use was also considered. The likelihood ratio test (LRT) was applied to test whether the additive polygenic effect in each analysis accounted for a significant component of the variation for the trait under study, after adjusting for the covariates. This test compares the likelihood of a full model (covariates, and additive polygenic and residual random components) with that of a nested model (covariates, and the residual component only). The LRT is asymptotically distributed as a mixture of 12χ02, and 12χ12.

The inclusion of covariates in the variance component model might influence the proportion of the phenotypic variance associated with the polygenic effect. Increased precision might be obtained if the heritability estimates increase with the covariate adjustments in comparison to the reduced or unadjusted model. But, when the heritability estimates decrease with the covariate inclusion, it means that the covariate explains a part of the familial relationship associated to the polygenic effect.

Risk factors were analyzed both as continuous and categorical variables. Dichotomized traits were defined according to the ATPIII criteria [13], as follows: truncal obesity was diagnosed when waist was more than 102 cm in men and more than 88 cm in women. High fasting glucose was defined when glucose levels were ≥110 mg/dL or with current use of anti-diabetic medication. The definition of low HDL cholesterol was considered when levels were < 40 mg/dL in men and < 50 mg/dL in women, high triglycerides ≥ 150 mg/dL, high LDL-c ≥ 130 mg/dL, high total cholesterol ≥ 200 mg/dL or use of lipid lowering medication. Hypertension was defined as mean systolic blood pressure of ≥ 140 mmHg and/or diastolic blood pressure of ≥ 90 mmHg or the current use of anti-hypertensive medication. Heritabilities for dichotomized traits were also estimated under the variance component model using SOLAR. Because the current medication information was absorbed into the trait's dichotomization criterion, Model 3 was not fitted to those traits.

## Results

### Descriptive statistics

We examined 1,712 individuals in 119 families, representing 13.5% of the entire city's population above 18 years. Because 38 of the ascertained families were composed of fewer than 3 individuals we have decided to use the remaining 1,666 individuals in 81 families for the current analysis. Family size varied from 3 to 156 individuals with a mean of 21 subjects per family. Data from 631 nuclear families were available, with a size ranging from one to 14 offspring. The number of generations per family varied from 2 to 4. Fifty-four percent of the ascertained families had 3 generations, and 45% of the families had 2 generations. The mean age was 44 years, with the range of 18 to 95 years. Women accounted for 56 percent of the study subjects. Seventy-six (4.2%) subjects were on lipid-lowering medications, 70 (4.0%) subjects were on hypoglycemic medications, and 405 (24.3%) subjects were on antihypertensive medications.

Mean fasting glucose, triglycerides, HDL-cholesterol, waist circumference and systolic (SBP) and diastolic (DBP) blood pressures are showed in Table 1. We observed that the prevalence of high fasting glucose was higher among women (20.9% female vs. 17.4% male). The same was observed with high waist circumference (45.1% female vs. 8.7% male), low HDL-cholesterol (34.3% female vs. 16.9% male), high LDL-c (28.0% female vs. 22.7% male), and high total cholesterol (36.2% female vs. 32.3% male). Hypertension was prevalent among both women and men (38.0% female vs. 33.8% male). Overweight and obesity were predominant among women, and this gender was more sedentary. For high triglycerides (28.6% female vs. 28.9% male), the values were similar in both genders. These variables were adjusted for the use of anti-diabetic and anti-hypertensive medication.

**Table 1:** Descriptive statistics of studied phenotypes, distributed by gender.

	**Total (n = 1,666)**	**Men (n = 723)**	**Women (n = 943)**
Age (years)	44.0 (16.9)	44.7 (17.5)	43.6 (16.5)
Waist circumference (cm)	87.4 (12.5)	86.8 (11.6)	87.9 (13.1)
Fasting glucose (mg/dL)	93.7 (30.0)	93.1 (26.5)	94.2 (32.0)
Triglycerides (mg/dL)	133.3 (75.4)	134.8 (82.6)	132.2 (69.3)
HDL-c(mg/dL)	55.9 (15.6)	53.9 (15.3)	57.5 (15.7)
Total cholesterol (mg/dL)	180.8 (48.0)	176.7 (48.5)	183.9 (47.4)
LDL-c (mg/dL)	98.7 (44.1)	96.0 (43.8)	100.7 (44.2)
Systolic blood pressure (mmHg)	126.8 (19.4)	130.3 (18.7)	124.1 (19.4)
Diastolic blood pressure (mmHg)	78.8 (11.4)	79.4 (11.6)	78.3 (11.2)
BMI (kg/m^2^)	24.4 (4.8)	23.4 (3.8)	25.2 (5.3)
High Fasting glucose(%)	19.4	17.4	20.9
High blood pressure (%)	36.2	33.8	38.0
Truncal obesity (%)	29.3	8.7	45.1
High Triglycerides (%)	31.1	30.6	31.5
Low HDL-c (%)	29.3	18.7	37.3
High LDL-c (mg/dL)	25.7	22.7	28.0
High TC (mg/dL)	34.5	32.3	36.2
BMI > 25 kg/m^2 ^(%)	38.6	29.9	45.2
BMI > 30 kg/m^2 ^(%)	12.7	6.4	17.5
Smokers (%)	16.6	20.6	13.5
Sedentarism (%)	21.1	17.8	23.7

Mean values and standard deviation for quantitative traits

Values for dichotomized traits are expressed in percentages.

### Heritability estimates

Heritability estimates for cardiovascular risk factor traits were all high, ranging from 26 to 51 percent (adjusted for age, sex, age^2^, age × sex), and BMI was the highest (Table 2). All heritability estimates were highly significant (*p *< 0.0001). Waist circumference, fasting glucose, triglycerides, LDL-cholesterol, BMI and systolic blood pressure showed excessive skewness and kurtosis and were therefore log-transformed. After the trait transformations, when the residual kurtosis remained too high the *tdist *procedure implemented in SOLAR was used (Table 2).

**Table 2:** Heritability (h2) estimates, and standard error, for each quantitative trait for cardiovascular risk factors (in percentage), unadjusted (Model 1), adjusted for age, sex, age2, and age × sex interaction (Model 2), and adjusted for age, sex, age2, and age × sex interaction, and current medication (Model 3).

	Model 1*	Model 2*	Model 3*
Waist circumference (cm)	26.1(0.042)	40.1(0.049)	-
Fasting glucose (mg/dL)^Δ^	32.8(0.046)	34.5(0.047)	32.3(0.047)
Triglycerides (mg/dL)	25.7(0.044)	28.8(0.048)	28.4(0.048)
HDL-cholesterol (mg/dL)	31.2(0.045)	32.0(0.046)	32.3(0.046)
Systolic blood pressure (mmHg)^Δ^	15.0(0.036)	25.9(0.042)	20.6(0.042)
Diastolic blood pressure (mmHg)^Δ^	16.4(0.037)	26.2(0.044)	21.0(0.044)
Total Cholesterol (mg/dL)^Δ^	28.6(0.047)	29.2(0.049)	30.4(0.049)
LDL-cholesterol (mg/dL)^Δ^	26.3(0.048)	26.3(0.049)	27.2(0.050)
BMI (kg/m^2^)	39.1(0.045)	51.0(0.048)	-

* p-value < 0.0001

Δ using tdist procedure in SOLAR

We performed quantitative genetic analyses for each trait, prior to and after adjustment for age, sex, age^2^, age × sex interaction, and current medication. The heritability estimates are shown in Table 2 and are expressed as the percentage of the total phenotypic variability due to the polygenic component, before and after adjustment for covariates. Before adjustment (Model 1), heritabilities of systolic and diastolic blood pressure were 15% and 16.4%, waist circumference 26.1%, triglycerides 25.7%, fasting glucose 32.8%, HDL-c 31.2%, TC 28.6%, LDL-c 26.3%, and BMI 39.1%. Adjustment for age, sex, age^2^, and age × sex interaction (Model 2) increased heritability estimates for all traits mainly SBP (25.9%), DBP (26.2%), waist circumference (40.1%), and BMI (51.0%). When we accounted for medication use (Model 3), heritability estimates were reduced for all traits apart from HDL-c, total cholesterol, LDL-c and triglycerides.

When the cardiovascular risk factors were treated as dichotomized traits, the estimates of heritability varied from 25.5 for high triglycerides to 54.5 percent for high fasting glucose after adjusting for covariates. (Table 3)

**Table 3:** Heritability (h2) estimates, and standard error, for cardiovascular risk factors treated as dichotomized traits, unadjusted (Model 1) or adjusted for age, sex, age2, and age × sex interaction (Model 2).

	**Model 1**	**Model 2**
Truncal obesity	25.44*	40.59*
High fasting glucose	45.90*	54.52*
High triglycerides	24.03*	25.50*
Low HDL-cholesterol	31.69*	37.79*
High Total-cholesterol	37.33*	38.08*
High LDL-cholesterol	37.09*	37.10*
Hypertension	13.24 (p = 0.0006)	32.49*

*p-value < 0.0001

## Discussion

The Baependi Heart Study provides an exceptional opportunity to characterize the genetic contributions to cardiovascular risk factors in a highly admixed population such as the Brazilian population. In this study we evaluated 1,666 individuals in 81 families to estimate the heritability of cardiovascular risk factors. These values were high, and varied from 26% (SBP and DBP) to 51% (BMI).

Although cardiovascular risk factors have been definitively demonstrated to be heritable, the heritability estimates of traits such as blood pressure levels, fasting glucose, lipids and obesity (3,4,7) are largely variable among populations and are dependent on the inclusion criteria used in a particular study (3,4). Nevertheless, despite these operational limitations, heritability attributed to cardiovascular risk factor traits is significant in most studies reported to date.

In this study the estimation of the genetic component of variance was limited to that attributable to polygenic effects and probably results from actions of more than one gene. If other nonadditive sources of genetic variation are considered, such as dominance or epistasis, then these observed heritabilities will represent lower bounds.

Conducting gene mapping studies in admixtured populations might be advantageous secondary to the possibility of finding genetic markers associated with a particular trait in a smaller linkage disequilibrium block. In this scenario fine-mapping of QTLs may prove to be more successful then identifying genetic determinants in populations were LD blocks span a significantly higher genomic interval.

It is difficult to compare our data with those from previous studies. The majority of studies were conducted in specific populations with different *a priori *risk for cardiovascular disease. The Framingham Heart Study was conducted in the general population, but blood glucose measurements were conducted in non-fasting samples. In addition, mean age was higher than the one observed in our population and only individuals 30 years old or higher were evaluated. In this study the observed heritability was high: systolic blood pressure (48%), fasting glucose (39%), triglycerides (56%), HDL-c (62%), and BMI (51%) [3]. The San Antonio Family Heart Study was designed to investigate the genetics of heart disease and its risk factors in Mexican Americans which are considered a high risk population because of the unusually high diabetes prevalence. In agreement with our estimates, heritability values as high as 46% of HDL-c variance, 42% of BMI variance, 40% of triglycerides variance, and 18% of systolic blood pressure and fasting glucose variance were observed [14]. The Northern Manhattan Family Study is a cohort of high risk Caribbean-Hispanic probands, in which inclusion criteria were defined by one of the following: reporting a sibling with a history of myocardial infarction or stroke; or having two of three quantitative risk phenotypes (maximal carotid plaque thickness, left ventricle mass divided by body surface area, or homocysteine level) above the 75^th ^percentile in the Northern Manhattan Study. The results of this study also showed high heritability for cardiovascular risk factors: HDL-c (60%), triglycerides (47%), waist circumference (46%), fasting glucose (24%), diastolic blood pressure (21%) and systolic blood pressure (16%) [15]. The Kiel obesity prevention study described heritabilities of 54% for truncal obesity, 39% for HDL-c, 31% for fasting glucose and triglycerides, 27% for DBP and 18% for SBP. The inclusion criterion for this study was the presence of at least one overweight or obese family member in a white Northern-European population [16].

It should be noted, however, that in spite of these caveats the heritability estimates from the present study are in the range of commonly reported heritability estimates from other populations (Table 4).

**Table 4:** Cardiovascular risk factor heritability estimates (percentage) in different studies.

	Studied population	Number of Individuals Studied	Number of ascertained Families	Waist circumference	Fasting glucose	Triglycerides	HDL-c	Total Cholesterol	LDL-c	Systolic blood pressure	Diastolic blood Pressure	Body Mass Index
Northern Manhattan Family Study [16]	Caribbean-hispanic. High cardiovascular risk individuals	803	89	46.0	24.0	47.0	60			16.0	21.0	
Covariates				Gender age	Gender, age, hypoglice mic drug	Gender, age, hypolipidemic drug	Gender, age, hypolipide mic drug			Gender, age, anti-hypertensive medication	Gender, age, anti-hypertensive medication	
San Antonio Family Heart Study [14]	Mexican-american individuals. High cardiovascular risk individuals	950	42	5.8 (WHR)	18.0	40.0	46.0			18.0		42.0
Covariates				Gender, age, age^2^, diabetes, diabetes duration, total dietary cholesterol ntake, menopausal status	Gender, age, age^2^, total protein.	Gender, age, age^2^, diabetes, polyunsaturated/sat rated fat intake, oral contraceptive use	Gender, total alcohol consuption, smoking status, postmenopausal estrogen use	Gender, age, age2, oral contraceptive use	Gender, age, age 2, menopausal status	Gender, age, age^2^, diabetes, caloric intake	Gender, age, age^2^, diabetes	Gender, age, age^2^, diabetes, diabetes duration, total dietary cholesterol intake,
Community-based study of healthy families [18]	Healthy individuals, northern European ethnicity	537	89		21.0	19.0	44.0		33.0	19.0		37.0
Covariates					Gender, age	Gender, age	Gender, age		Gender, age	Gender, age		Gender, age
Chin-Shan community family study [26]	Chinese individuals. General population, mainly young individuals	1356	-	17.0	27.2	26.7	29.9	32.8	40.1	32.1	23.2	21.6
Covariates	Not informed											
The Kiel obesity prevention study [16]	Northern European Caucasian with at least one overweight or obese individual in the family	492	86	54.0	31.0	31.0	39.0			18.0	27.0	
Covariates				Gender, age	Gender, age	Gender, age	Gender, age			Gender, age	Gender, age	
Framingham Heart Study [3]	General population	1617	-		39.0 (non-fasting)	56.0	62.0			48.0		51.0
Covariates					Gender, age	Gender, age	Gender, age			Gender, age		Gender, age
**Strong Heart Family Study [20]**	Native american individuals. High cardiovascular risk individuals	950	32		29.0	40.0	50.0			23.0	34.0	44.0
Covariates					Gender, age, alchool consuption, estrogen use	Gender × age	Gender, age, alcohol consuption, estrogen use			Gender, age, gender × age	Gender, age, alcohol consuption, estrogen use	
**Baependi Heart Study**	**Brazilian individuals. General population**	**1666**	**81**	**40.1**	**34.5**	**28.8**	**32.0**	**29.2**	**26.3**	**25.9**	**26.2**	**51.0**
**Covariates**				Gender, age, age^2^, gender × age	Gender, age, age^2^, gender × age	Gender, age, age^2^, gender × age	Gender, age, age^2^, gender × age	Gender, age, age^2^, gender × age	Gender, age, age^2^, gender × age	Gender, age, age^2^, gender × age	Gender, age, age^2^, gender × age	Gender, age, age^2^, gender × age

Another potential problem in comparing the various studies is the different set of covariates used in each final model. In addition, some studies excluded individuals with vascular disease or diabetes mellitus [17,18].

In our data, heritability estimates increased for all traits after adjustment for sex and age, and model adjustment was particularly important for the final estimate for waist circumference, SBP, DPB and BMI. Interestingly, for fasting glucose, DBP, and SBP, unlike the results observed in the adjustment for age and gender (Table 2, Model 2) we observed an important reduction in the estimated heritability when prior use of medication was included as a covariate in the model (Table 2, Model 3). This means that medication use for those four traits contains information about trait variability due to the polygenic component, rather than to the error component. Such patterns of effect suggest that, in our population, the current use of anti-hypertensive and anti-diabetic medications, but not the use of lipid lowering medication, is associated with the estimated heritability and, consequently, with genetic determinants. In fact, the relationship between covariates and variance components spaces have been explored in linkage studies [19]. One possible explanation for this fact is that being on treatment is a marker for unusual values of the trait, and hence explains some of the genetic variation.

There are potential shortcomings and limitations that must be considered when generalizing about our findings. Firstly, we only used a single measurement in time for the present analysis. It is possible that several cardiovascular risk measurements taken over time may provide additional information and represent more stable estimates. In fact, this has been shown for long-term blood pressure heritability estimates [20], and may indeed have a more pronounced effect for other measured traits that are notoriously variable (for example; triglycerides). In addition, our results for the dichotomized traits are limited in at least two ways. First, it is known that the dichotomization of continuous variables result in loss of information. Second, the results obtained are intrinsically dependent on the cut-off values adopted in our analysis. In particular, this may have a profound impact on the estimates of waist circumference and low-HDL cholesterol that use cut-off values that were operationally determined for the metabolic syndrome diagnosis and established mainly in Caucasian populations [4,16,21]. It should be noted, however, that different proposals for these cut-off values do exist and vary between different diagnostic criteria [22-24], different populations [4,15-18,21,25-31] and different genders [28,32]. Clearly, using alternative cut offs would lead to different heritability estimates and this point should be better explored in future studies. Nevertheless, we believe obtaining heritability estimates for clinically used dichotomized traits is important for study comparison and future mapping efforts and algorithm construction. Finally, one can not differentiate common environmental from pure genetic factors in this study. Familial aggregation of cardiovascular risk factors has complex interactions among genes and environmental factors.

## Conclusion

Taken together, we provide the first evidence that a significant proportion of the variability of cardiovascular risk factors is explained by genetic factors in this large sample of a Brazilian population. Further studies of genetic linkage and candidate gene association are warranted to identify specific genetic variants associated with these important predictors of cardiovascular disease events and mortality.

## Competing interests

The authors declare that they have no competing interests.

## Authors' contributions

CMO and ACP performed the field work. CMO, ACP, JP and MA performed the statistical analyses. CMO and ACP drafted the manuscript under the supervision of JP, MA and JEK. ACP, JP and JEK supervised the study. All authors read and approved the final manuscript.

## Pre-publication history

The pre-publication history for this paper can be accessed here:


